# Dual Coronary-Pulmonary Artery Fistula in a Patient with Severe Bicuspid Aortic Valve Stenosis

**DOI:** 10.14797/mdcvj.1187

**Published:** 2023-04-10

**Authors:** Rody G. Bou Chaaya, Yasser Sammour, Samarthkumar Thakkar, Ziad Jaradat, William J. Gill, Omar Batal

**Affiliations:** 1 Houston Methodist DeBakey Heart & Vascular Center, Houston, Texas, US; 2 Indiana University School of Medicine, Indianapolis, Indiana, US

**Keywords:** bicuspid aortic valve, aortic stenosis, coronary artery, fistula, pulmonary artery

## Abstract

A 62-year-old male presented to the emergency department with acute viral bronchitis and worsening of his chronic dyspnea on exertion. Incidentally, a murmur was detected on physical examination. Extensive work-up, including coronary computed tomography angiography, revealed a rare combination and potential association between severe bicuspid aortic valve stenosis and coronary-pulmonary artery fistulas.

## Background

Coronary artery fistulas (CAFs) are rare vascular abnormalities that can be congenital or acquired throughout life.^1^ Congenital CAFs are associated with other heart abnormalities in around 30% of the cases.^2^ Since few cases of coronary-pulmonary artery fistula (CPAF) have been recorded, it’s likely that a congenital association exists with the bicuspid aortic valve. We report the case of a 62-year-old male presenting with acute bronchitis and worsening chronic dyspnea on exertion. Incidentally, he was found to have dual coronary-pulmonary artery fistula, ascending aortic ectasia, and severe bicuspid aortic valve (BAV) stenosis.

## Case Presentation

A 62-year-old man with no previously established care presented to the emergency department with complaints of fever, productive cough, left lower chest pain, and shortness of breath. He reported exertional chest pain and dyspnea that had progressively worsened before this acute episode. Physical examination findings were as follows: blood pressure 111/77 mm Hg; heart rate 107 beats/minute; respiratory rate 20 breaths/minute; temperature 38.2°C; oxygen saturation 94% on 2 L O2; and a 3/6 harsh late-peaking systolic ejection murmur was heard across the precordium. Bilateral rhonchi were present. Mild bilateral leg swelling was noted. He was admitted for management of acute shortness of breath and further workup of chronic dyspnea of exertion.

He had no past medical history of heart or lung disease or other known comorbidities. His family history was also negative for known heart disease.

## Investigations

A 12-lead electrocardiogram showed sinus rhythm and signs of left ventricular hypertrophy with nonspecific repolarization abnormalities. Troponin T was 154, 152, and 146 ng/L (3-hour intervals, normal range 0-19 ng/L) and BNP was 327 pg/mL (normal range 0-100 pg/mL). Procalcitonin was 0.11 ng/mL (normal range < 0.15 ng/mL). Blood cultures, pneumococcal, and legionella urinary antigen tests were negative. COVID-19 RT-PCR test was negative. A respiratory viral panel PCR was completed and came back positive for respiratory syncytial virus A (RSV-A). A chest x-ray showed cardiomegaly with pulmonary vascular congestion and small bilateral pleural effusions.

**Figure 1:**
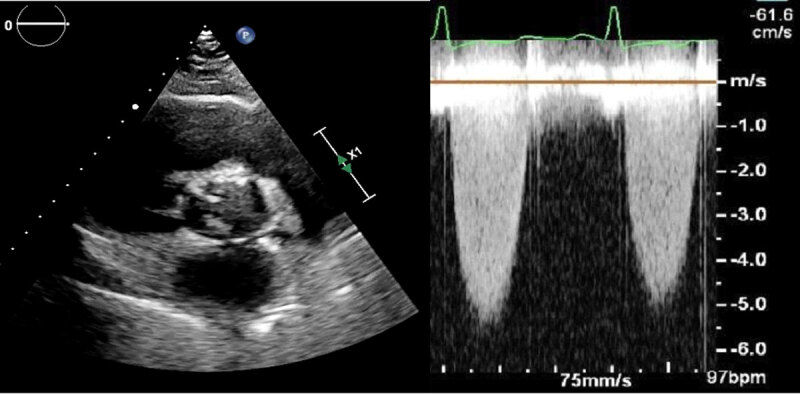
Echocardiogram of short axis view showing a bicuspid valve with increased velocity across the valve; see also at https://youtu.be/4C2kncWIOqM.

A chest computed tomography (CT) scan pulmonary embolism protocol was negative for pulmonary embolism. It also confirmed the x-ray findings and noted bronchial wall thickening consistent with bronchitis. Transthoracic echocardiography was performed and showed severe aortic stenosis associated with bicuspid aortic valve. On echocardiogram, the aortic valve area was 0.84 cm^2^, the mean aortic valve gradient was 75 mm Hg, and the velocity across the valve was 5.3 m/sec (Figure 1, Videos 1, 2). Left ventricular ejection fraction was 35% with mild concentric left ventricular hypertrophy.

**Video 1:** Echocardiogram of short axis view showing a bicuspid valve with increased velocity across the valve; see also at https://youtu.be/4C2kncWIOqM.

**Video 2:** Echocardiogram of long axis color Doppler view showing a bicuspid valve with severe stenosis; see also at https://youtu.be/Y4cAeFUFoPs.

The patient received supportive treatment for his acute bronchitis and intravenous diuretics, which markedly improved his clinical condition. He was discharged on day 7 and was started on oral Lasix, aspirin, statin, metoprolol succinate, and a low-sodium diet. He was seen by cardiology and referred for surgical aortic valve replacement (SAVR) given the severity of his BAV stenosis.

**Figure 2:**
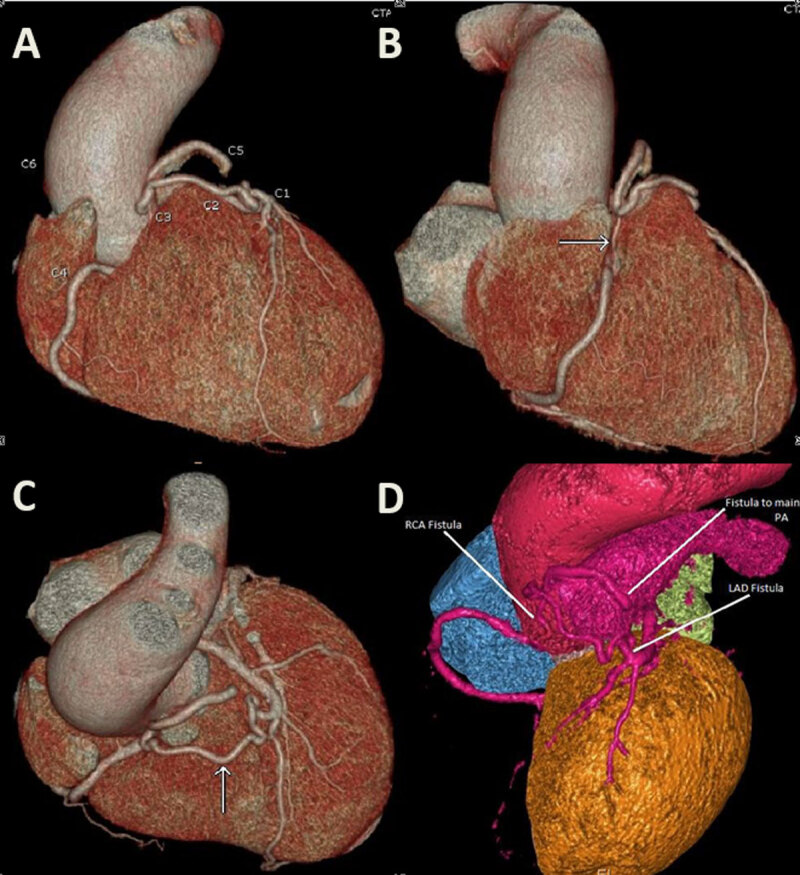
A coronary computed tomography angiography 3-dimensional (3D) reconstruction showing a coronary artery fistula arising from the left anterior descending artery joining another fistula arising from the right coronary artery, both draining into the main pulmonary artery. (A) C1: left anterior descending artery; C2: left anterior descending artery fistula; C3: right coronary artery fistula; C4: right coronary artery; C5: drainage into the pulmonary artery; C6: ectasia of the ascending aorta; (B) arrow: fistula connecting to the right coronary artery; (C) arrow: fistula connecting to the left anterior descending artery; (D) 3D reconstruction of the fistula excluding the right ventricle.

**Figure 3:**
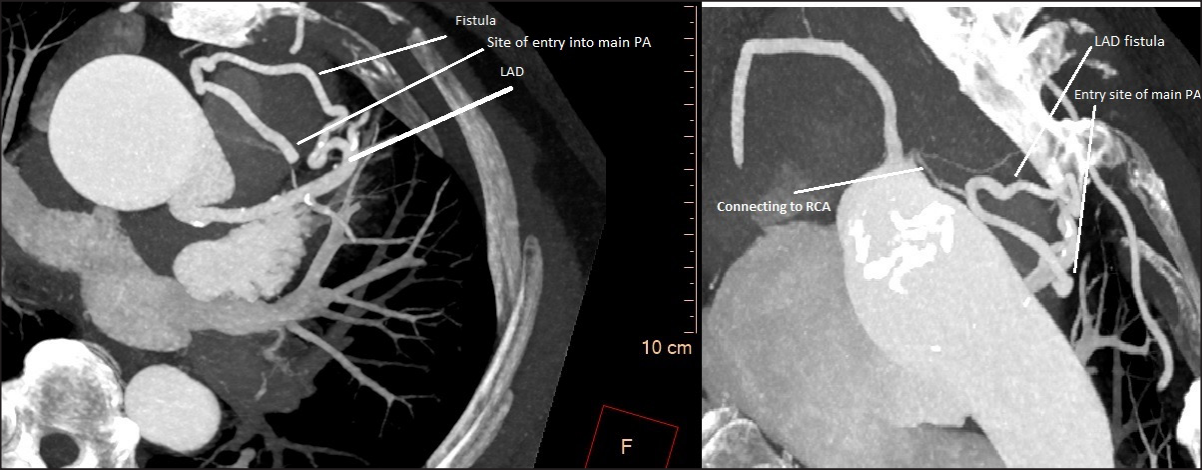
A coronary computed tomography angiography axial view showing the course of the fistula.

In preparation for an aortic valve replacement, a coronary angiography was performed. Two coronary fistulas were identified: one arising from the mid left anterior descending (LAD) and one from the proximal right coronary artery (RCA) (Videos 3, 4). Also, 50% coronary artery stenosis was observed distal to the fistula in mid-LAD. Several views failed to visualize the course of the fistulas. Therefore, a coronary CT angiography (CCTA) was performed to better identify the course. As shown in Figures 2 and 3, the mid-LAD fistula connects to the proximal RCA fistula at the cardiac base anterior to the aortic root. They both drain into the main pulmonary artery via a single connection. In addition, the ascending aorta was moderately dilated in diameter (45 mm), and the mid-LAD showed a mixed, mostly noncalcified plaque with 60% luminal stenosis.

**Video 3:** Coronary angiography of fluoroscopy showing a coronary artery fistula arising from the proximal right coronary artery; see also at https://youtube.com/shorts/yZ3eEaNSuPw.

**Video 4:** A coronary angiography of fluoroscopy showing a coronary artery fistula arising from the mid-left anterior descending artery and draining into the main pulmonary artery; see also at https://youtube.com/shorts/gHFhO8Gxb8c.

## Differential Diagnosis

The differential diagnosis of acute on chronic dyspnea includes, but is not limited to, acute decompensated heart failure, acute viral bronchitis, undiagnosed valve disease (including severe aortic stenosis), acute community acquired pneumonia, and pulmonary embolism. The presence of sepsis and the incidental detection of a murmur on physical exam prompted admission to the hospital for further investigation.

## Outcome and follow-up

The patient presented later with decompensated heart failure and underwent successful transcatheter aortic valve replacement, after which his functional capacity markedly improved.

## Discussion

This case describes a patient who presented with acute RSV-A bronchitis and acute chronic dyspnea on exertion. He was found to have new-onset heart failure with reduced ejection fraction 35%, severe BAV stenosis, ascending aortic ectasia, 50% mid-LAD stenosis, and a dual coronary-pulmonary artery fistula.

BAV is the most common congenital cardiac abnormality, affecting 1% to 2% of the population.^3^ Dilatation of the proximal aorta is present in up to 50% of patients with BAV.^4^ A moderate dilation of the aorta (45 mm) was present in our patient.

A CAF is a rare coronary artery anomaly found in 0.3% to 1.3% of patients undergoing diagnostic coronary angiography.^1^ It has a prevalence of 0.9% on CCTA, with drainage into the pulmonary arteries ranging from 0.17% to 0.68%.^2^ Approximately 50% of CAFs originate from the RCA and more than 90% drain into the venous circulation, giving rise to a left-to-right shunt. Dual CAF involving both right and left coronary arteries is uncommon, accounting for only 5% of all cases.^5^ The most common manifestations of these anomalies are angina (due to the “coronary steal” phenomenon), congestive heart failure, arrhythmias, and thrombosis. In general, younger patients are asymptomatic while older individuals commonly develop symptoms.

In 20% to 45% of cases, congenital CAFs are associated with other heart abnormalities including atrial septal defect, patent ductus arteriosus, ventricular septal defect, pulmonary atresia, and tetralogy of Fallot.^2,5^ Since few cases of CPAF have been recorded, it is likely that a congenital association exists with the bicuspid aortic valve. Singhal et al. described the case of a 67-year-old who presented with chest pain and reduced exercise tolerance. Further investigations showed congenital left main coronary artery to main pulmonary artery fistula associated with moderate BAV stenosis. A CCTA was necessary to visualize the course of the fistula. The patient was treated with SAVR and ligation of the fistula, with resolution of symptoms at 6 months.^6^ Other cases report the combination of CAF to the aorta, left atrium, right ventricle, and BAV, either isolated or in addition to other congenital anomalies.^7,8^

Another explanation for the association of BAV and CPAF is that in the setting of severe aortic stenosis secondary to BAV, an acquired deficiency in von Willebrand factor (vWF) results in a lack of vascular integrity, allowing fistulae to form. This would be comparable to the angiodysplasia seen in Heyde syndrome.^9^ Alammar et al. reported the case of a patient presenting with heart failure due to severe aortic stenosis, with CCTA done for better anatomical delineation after an angiogram showed multiple coronary arterial-venous malformation with multiple coronary artery fistulae connected to the right pulmonary artery.^10^ Also, Liu et al. described the case of a patient with von Willebrand disease who presented with chest pain and was found to have RCA CAF to the left atrium.^11^ Both cases support the hypothesis of a potentially acquired vWF disease contributing to the pathogenesis.

In symptomatic patients, surgical ligation or percutaneous transcatheter embolic occlusion is highly recommended, while management is controversial for patients without symptoms.^12^ If the patient needs a surgical procedure for concomitant disease, surgical closure of fistula remains the treatment of choice.

This case emphasizes the utility of CCTA in patients with complex cardiovascular anatomy. Two of the previously mentioned cases required CCTA to better characterize the vascular anatomy and guide surgery. A recent study shows that CCTA is preferred over invasive coronary angiography (ICA) in detecting CPAF. In a cohort of CPAF identified by CCTA, 25 patients had a concomitant ICA, which showed CPAF in only 20 of them.^13^

Our patient had a 50% stenosis distal to the fistula in the mid-LAD. Although this is likely primary atherosclerosis, it is important to note that increased flow through the coronaries in the setting of fistula has been associated with early coronary artery disease.^14^

Finally, angina in this case could be related to the fistula, the LAD lesion, or both, and management could be challenging. Episodic steal phenomenon that occurs with exertion often yields negative results with vasodilator stress tests due to the poor vasodilator capacity of the fistula and the increased flow in the original nutrient vessel.^15^ Also, fractional flow reserve or instant wave-free ratio may yield controversial results with two consecutive lesions. It is likely that the 50% mid-LAD lesion is rendered more hemodynamically significant by the preobstruction diversion of blood through the fistula.

## Key Points

- Detection of a murmur, in the right clinical setting, should prompt further investigation.
- Although a rare combination, there appears to be a potential association between coronary-pulmonary artery fistula and bicuspid aortic valve.
- Coronary computed tomography angiography is preferred over invasive coronary angiography in the diagnosis of coronary-pulmonary artery fistula. It is an excellent tool that detects congenital anomalies in the setting of bicuspid aortic valve, characterizes the vascular anatomy, and guides surgery.
